# A Defined Methodology for Reliable Quantification of Western Blot Data

**DOI:** 10.1007/s12033-013-9672-6

**Published:** 2013-05-25

**Authors:** Sean C. Taylor, Thomas Berkelman, Geetha Yadav, Matt Hammond

**Affiliations:** 1 Bio-Rad Laboratories (Canada) Ltd, 1329 Meyerside Dr, Mississauga, ON L5T 1C9 Canada; 2 Bio-Rad Laboratories, 6000 Alfred Nobel Drive, Hercules, CA 94547 USA; 3 Bio-Rad Laboratories, 2000 Alfred Nobel Drive, Hercules, CA 94547 USA

**Keywords:** Western blot, Densitometry, Protein expression

## Abstract

Chemiluminescent western blotting has been in common practice for over three decades, but its use as a quantitative method for measuring the relative expression of the target proteins is still debatable. This is mainly due to the various steps, techniques, reagents, and detection methods that are used to obtain the associated data. In order to have confidence in densitometric data from western blots, researchers should be able to demonstrate statistically significant fold differences in protein expression. This entails a necessary evolution of the procedures, controls, and the analysis methods. We describe a methodology to obtain reliable quantitative data from chemiluminescent western blots using standardization procedures coupled with the updated reagents and detection methods.

## Introduction

Western blotting has been a staple in life science labs for several decades—ever since researchers published the first detailed description of this protein detection technique in 1979 [1]. This multistep method determines the presence or absence, size, and modification or degradation states of target proteins, as well enables the quantitation of proteins from complex protein mixtures or homogenates [2–4]. However, there are many potential stumbling blocks in this procedure that can preclude reliable results. These include challenges related to every step of the western blotting procedure, from sample preparation, normalization, SDS–PAGE gel loading, protein transfer, primary and secondary antibody selection, incubations, and washes, detection method selection to densitometric analysis. A recent report predicts that approximately 25 % of the accepted papers include at least one inappropriately manipulated figure and many of these are associated with western blotting [5]. This underlines the negative perception by which the scientific community views the western blot data. Thus, editors and reviewers of scientific journals are looking at western blot results, particularly at the densitometric analysis to determine the fold differences in protein expression, with greater scepticism, often requesting the raw data files.

Chemiluminescent western blot data, derived from film-based detection, poses distinct challenges in producing quantifiable, reproducible data. These problems stem from a low-dynamic range of detection and the difficulty in accurately determining the limit of detection [6, 7]. The scientific community has largely ignored these challenges, mostly because of a common misperception that film produces the highest quality data from western blots. However, unless the experiments are performed with a deep understanding of these limitations, this method of detection is an approximation at the best and often nonquantitative if used inappropriately. By contrast, the rapid evolution of affordable and highly sensitive gel and blot imaging technologies coupled with new reagents gives researchers the means to produce truly quantitative western blot data—as long as the process is carried out with proper technique, validation, and controls. These new tools and techniques eliminate the limitations associated with film-based detection and meet the journal reviewers’ demands for quantifiable protein expression data.

Here, we will demonstrate how standardized protein samples, when processed with film versus digital imaging methods and different normalization approaches, produce vastly different results. Based on our findings, we propose a rigorous and simple methodology to produce high quality, reproducible, and quantitative western blot data.

## Materials and Methods

### Protein Sample Preparation and Separation

A mixture containing a lysate from HeLa cells and purified yeast alcohol dehydrogenase (ADH) (Sigma Aldrich) in Laemmli buffer (Bio-Rad) was used as the starting material for separation on Criterion TGX AnykD Stain-Free gels (Bio-Rad). All sample wells were loaded with 20 μl of the protein mixture with separation using the Criterion Dodeca gel apparatus (Bio-Rad) for 1 h at 200 V.

### Gel Imaging

All gels were imaged using the stain-free application on the ChemiDoc MP (Bio-Rad) imager immediately after the protein separation and prior to western blotting.

### Western Blot Transfer and Total Protein Imaging

Protein gels were blotted using the Trans-Blot Turbo transfer apparatus and PVDF Midi transfer packs (Bio-Rad). Membranes were immediately transferred to a blocking buffer for fluorescent western blotting (Rockland), and incubated with a gentle agitation for 1 h at room temperature. During blocking and after uniform wetting in blocking buffer, the membranes were imaged for the total protein transferred using the stain-free application on the ChemiDoc MP imager.

### Antibody Incubation and Chemiluminescent Detection

Membranes were incubated overnight with gentle agitation at 4 °C in 30 ml of blocking buffer with a mixture containing anti-yeast ADH rabbit polyclonal Ab (ABCAM) (1:5000 dilution) and anti-human GAPDH mouse monoclonal Ab (Rockland) (1:10000 dilution). These blots were washed five times for 3 min in Tris-Buffered Saline with Tween-20 (TBST; 500 mM NaCl, 20 mM Tris–Cl, pH 7.5, 0.05 % (w/v) Tween 20), and incubated in 40 ml of a mixture containing goat anti-mouse HRP Ab (Bio-Rad) (1:50000 dilution) and goat anti-rabbit HRP Ab (Bio-Rad) (1:50000 dilution) in blocking buffer for 1 h with gentle agitation at room temperature. This was followed by five 3-min washes in TBST at room temperature and incubation in Clarity western ECL substrate chemiluminescent detection reagent (Bio-Rad) for 5 min prior to image acquisition.

### Blot Imaging and Densitometric Analysis

**Fig. 1:**
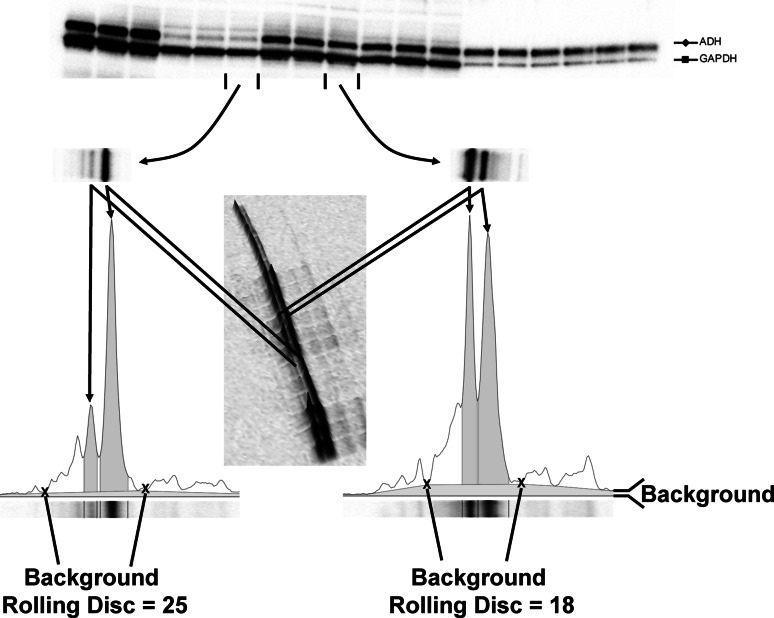
Image acquisition and densitometric analysis. ImageLab software version 4.1 (Bio-Rad) was used for image acquisition and densitometric analysis of the gels, blots, and film in this study. The software interprets the raw data in three dimensions with the length and width of the band defined by the “Lanes and Bands” tool in concert with the “Lane Profile” tool such that the chemiluminescent signal emitted from the blot is registered in the third dimension as a peak rising out of the blot surface. The density of a given band was measured as the total volume under the three-dimensional peak, which could be viewed in two dimensions using the “Lane Profile” tool to adjust the precise width of the band to account for the area under the *shaded* peak of interest. Background subtraction was set by using the rolling disc setting in the “Lanes” tool. The rolling disc values were set such that the background was subtracted under the band (i.e., peak) of interest in a uniform manner between the lanes of a given blot. In this case, the rolling disc for the two lanes analyzed was set to 18 and 25, respectively, such that the peaks of interest were cut at a consistent level between the markers shown with an “X”

The chemiluminescent blots were imaged first with the ChemiDoc MP imager (Bio-Rad) and then on film. Films were subsequently imaged with the ChemiDoc MP using the white light conversion screen and the silver stain (visible stain) application. The Band Analysis tools of ImageLab software version 4.1 (Bio-Rad) were used to select and determine the background-subtracted density of the bands in all the gels and blots (Fig. 1). For background (called rolling disc in the software) subtraction, a value of 1 was used while imaging the gel and blot images for the total protein measurements from the lanes, while for the film and imager data acquired from the chemiluminescent blots, a rolling disc between 10 and 25 was used to ensure a consistent peak cutting for densitometric analysis (Fig. 1).

## Results and Discussion

For most analytical techniques, the key to obtain accurate and reproducible results is in understanding the limits of the tools employed and defining the lower and upper limits of quantitation and the linear dynamic range. Measurements using standard curves remain the best method for accurately defining these parameters, and are typically performed using serial dilutions of a representative sample [8, 9]. The requirement for clean and interpretable data is critical for determining these two parameters. Western blotting involves the following complex series of steps and obtaining quantifiable results requires that all the steps be performed rigorously as most of them are interdependent:

1. Lysis of cells or tissue
2. Quantification of lysate total protein concentration
3. Equal loading and separation of samples using SDS–PAGE
4. Complete transfer of proteins separated on the gel to nitrocellulose or PVDF membrane
5. Determination of proper dilutions for primary and secondary antibodies
6. Optimal detection of the chemiluminescent signal
7. Quantification of densitometric data

Thus, a methodology to test and confirm the quality of each step should be employed.

### Validation of the Loaded and Transferred Proteins

The handling, storage, and lysate preparation of tissue and cell culture specimens can be complicated by the nature of the sample itself and by the large array of reagents and equipment available to prepare them. Careful consideration should be given to determine the best approach to prepare samples in order to get a reliable end result. Since most lysis buffers contain detergents such as Triton X-100 or sodium dodecyl sulphate (SDS), a detergent-compatible protein assay should be chosen.

**Fig. 2:**
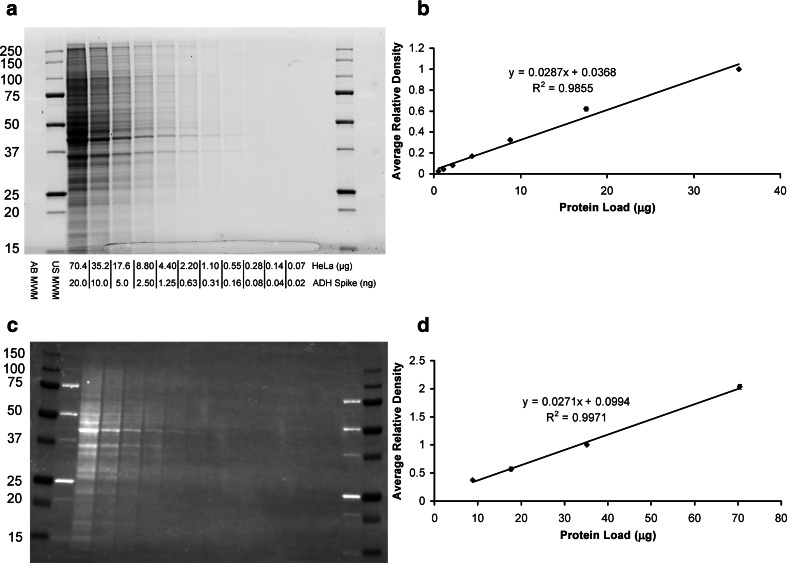
Western blot validation with stain-free gel technology. **a** and **c**: Images obtained from ChemiDoc MP imager of the gel (**a**) and transferred blot (**c**) from a two-fold dilution series of a HeLa cell lysate with spiked-in ADH protein. **b** and **d**: Average relative lane density of the total protein load of three gels (**b**) and the associated blots (**d**) to determine the linear dynamic range for stain-free detection. Molecular weight markers were run in the first and last two lanes of the gel. AB MWM and US MWM are the Precision Plus All Blue and Unstained molecular weight markers, respectively (Bio-Rad). *Error bars* represent the standard errors of the mean for three gels and associated blots

The typical amount of lysate loaded per lane of an SDS–PAGE gel is between 10 and 80 μg, often times, with the same amount of protein per lane loaded regardless of the antibody used or target probed. It is also a common practice to run the gel and then immediately transfer the separated proteins to PVDF or nitrocellulose membrane without verifying the in-gel consistency of protein loading or of the quality of separation. In this study, the stain-free gel technology (Bio-Rad) was used to image the SDS–PAGE separation of a two-fold dilution series of a HeLa cell lysate prior to transfer (Fig. 2a). The quantitative analysis of the total protein loaded per lane gave an excellent correlation (*R* ^2^ = 0.9855) to the two-fold dilutions over a linear dynamic range between about 1 and 35 μg of the loaded protein (Fig. 2b).

Following the transfer, the membranes were imaged to assess the transfer efficiency and lane-to-lane consistency of the transferred proteins, which retain the fluorescence imparted by the stain-free imaging process (Fig. 2c). Although, the linear dynamic range was much narrower for the transferred protein, a very good correlation (*R* ^2^ = 0.9971) to the two-fold dilutions was obtained in the typical range (between about 10 and 70 μg) of total protein load for most western blotting techniques (Fig. 2d). The reduced sensitivity of the fluorescent signal from the membrane versus the gel is the result of some protein transferring through the membrane (over-transfer) and much higher background due to the autofluorescence of the PVDF membrane under UV illumination during stain-free imaging. The combined effects of over-transfer and variable transfer efficiency support the use of densitometric data produced from the membrane for normalization as opposed to the gel.

### The Quantitative Linear Dynamic Range: Contrasting Detection Platforms

**Fig. 3:**
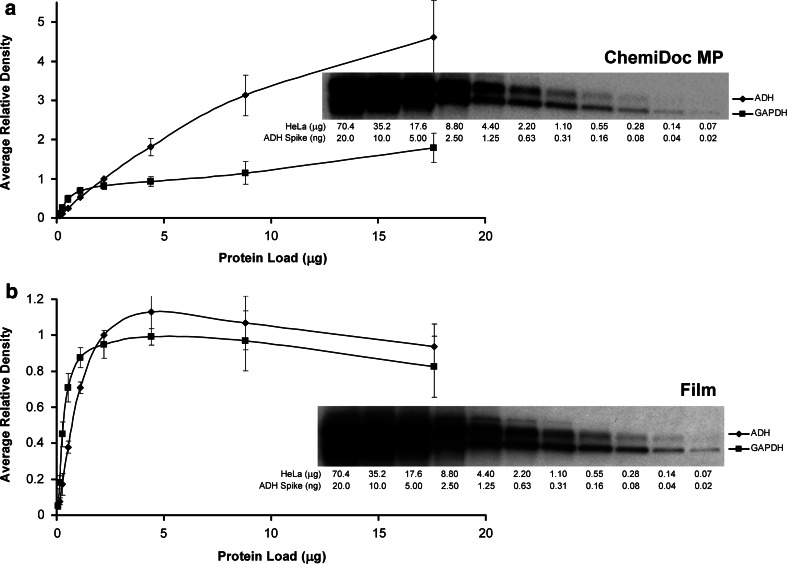
Defining the linear dynamic range of western blot detection. The chemiluminescent western blot of the two-fold dilution series of the HeLa lysate with spiked-in ADH protein from Fig. 2 was imaged with the ChemiDoc MP (**a**) and then with film (**b**). Blotting was performed using a mixture of rabbit- and mouse-derived primary antibodies to ADH and GAPDH, respectively, with the associated mixture of HRP-conjugated secondary antibodies. The average relative density and the standard error of the mean of the imaged bands are plotted against the actual protein load from four blots. The *upper* and *lower bands* denote ADH and GAPDH proteins, respectively

**Fig. 4:**
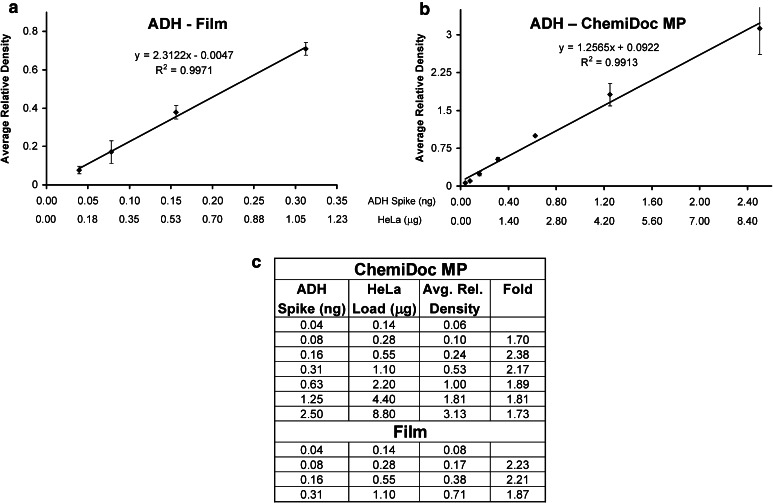
Contrasting the linear dynamic range of film-based and imager-based detection for ADH. A linear dynamic range of four dilutions for film (**a**) and seven dilutions for the ChemiDoc MP imager (**b**) was derived from the dilution series data in Fig. 3. The fold difference in densitometric data within each linear dynamic range correlated to the two-fold dilution series loaded on each blot (**c**)

Film has been the traditional method of choice for the detection of chemiluminescent western blots using a wide variety of detection reagents and horseradish peroxidase (HRP)-conjugated secondary antibodies. Although, film provides excellent resolution and sensitivity, the dynamic range of quantitation is poor [7]. On the other hand, with the next generation camera-based detection methods, both the sensitivity and linear dynamic range are excellent, which permits much more accurate quantification of the relative density between samples. This is illustrated in the comparison of image data for a two-fold dilution series of ADH generated from the same blot using film and camera-based detection methods (Fig. 3a, b). Here, a linear dynamic range of four dilutions (16-fold) between 0.04 and 0.31 ng was observed for ADH with film as opposed to the seven dilutions (128-fold) between 0.04 and 2.5 ng for the ChemiDoc MP imager (Fig. 4).

For the endogenous protein GAPDH, both film and the ChemiDoc MP were only linear for the lowest three dilutions. This can be attributed to very high abundance of this protein in the HeLa cell lysate (Fig. 3). In this case, more dilutions and a longer exposure time would extend the linear dynamic range, but these data show that to use GAPDH as a reliable and quantifiable loading control, a total protein load of not more than about 0.5 μg is required (Fig. 3).

For the film images, a slight decline in the density was observed with increasing protein load within the plateau region (Fig. 3b). This can be attributed to the saturation of background between and around the bands of interest. Given that the ADH and GAPDH bands of interest are already fully saturated at 4.4 μg of total protein load, the contribution of the increasing background density (that saturates quickly with the film) results in a net decrease in background-subtracted density for these proteins.

### Optimization of Protein Loading for Quantitation

All blot and gel detection systems generate a two-dimensional image, and signal intensity forms the third dimension of information (Fig. 1). Therefore, if excessive protein is loaded in the gel lane such that the width of the gel has been filled, the detector (whether film, camera, or scanner) will only capture the signal from the protein that resides near the surface of the gel. The same effect is observed during blotting where the protein transferred from an overloaded gel lane will form a layer on top of the protein already bound to the surface of the transfer membrane such that the primary antibodies will only bind to the surface layer of the transferred protein [10, 11]. The plateau observed for the same blot using both film- and camera-based detection is not due to the saturation of the detector, but rather is the result of protein saturation within the gel itself and consequently of the blot itself with the multiple layers of target protein bound to the membrane (Fig. 3).

Since many labs typically load a specific, fixed amount of protein (typically between 10 and 50 μg) into the gel lanes without determining the optimal protein load, there is a strong potential for gel saturation, particularly for loading controls such as GAPDH, beta-actin, and tubulin. Although, this results in consistent band densities among the sample lanes, these data are often the result of overloading the gel for these abundant proteins such that the densities obtained are far outside the linear dynamic range in the plateau. The best way to avoid this issue is to produce a standard curve from a two-fold serial dilution series over 12 dilutions starting from about 80 μg of a pooled lysate from representative samples across the experimental conditions [8]. A separate standard curve of band density versus protein load should be run to validate each primary antibody for western blot as shown in Fig. 3. In this fashion, the linear dynamic range of detection for each antibody can be determined, and the associated dilution factor required for individual sample loading can be obtained by diluting the samples to the mid point of the standard curve. In the present case, we determined that the linear dynamic range of ADH is between 0.04 and 2.5 ng of the purified protein (Fig. 4b, c).

### Loading Controls for Quantitation

The transfer efficiency of protein to a blotting membrane can be inconsistent across the gel, resulting in a gradual two- to four-fold increase or decrease in the signal between the lanes. Furthermore, the preparation and quantitation of sample lysates for the concentrations coupled with their physical pipetting into the lanes of a protein gel can also lead to inconsistent densitometric data. Loading controls are useful to normalize these technical artifacts and become increasingly important when measuring small differences in protein expression between samples.

The most common loading controls include housekeeping proteins, such as GAPDH, beta-actin and tubulin, which are constitutively expressed proteins that maintain cell viability. However, these proteins are generally highly expressed in samples and are frequently overloaded in the gel lane with the target protein such that they would not serve to normalize the loading [12, 13]. Although, the densitometric data would be consistent, this data could be an artifact of overloading as observed in our experiments where GAPDH levels were reaching a plateau above 0.5 μg of HeLa lysate (Fig. 3). Furthermore, the housekeeping proteins themselves can be variably expressed between the experimental conditions, thereby eliminating their usefulness for the normalization of western blots [14–17]. In order to avoid these issues, the total lane density of transferred protein on the membrane is being used for the normalization in lieu of the traditional loading controls [18–22].

**Fig. 5:**
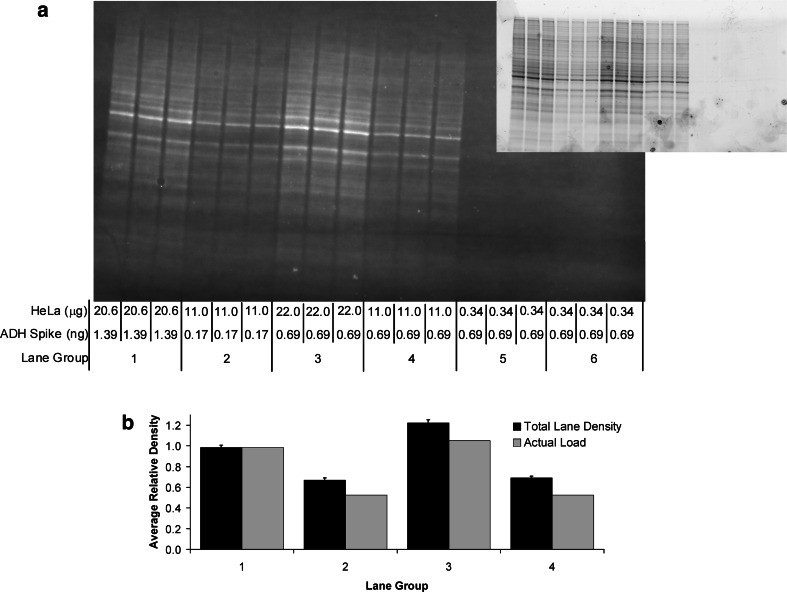
Verification and validation of western blotting. Four stain-free gels were loaded with measured amounts of HeLa lysate and spiked-in ADH. After separation, the gels were imaged to verify consistent loading (**a**, inset). The gels were then blotted and the respective blots imaged to validate the transfer efficiency and total lane density for normalization (**a**). The average relative density of total protein load (as detected from the stain-free fluorescence of the transferred protein in the blots) was compared to the relative difference in μg quantity of HeLa total protein load between the triplicate replicates of each lane group over four blots (**b**). A positive Pearson Correlation was obtained between total protein load and average relative density of transferred protein (*p* value of 0.0398)

Since the stain-free gel technology produced accurate and quantitative densitometric data from the captured fluorescence intensity of the transferred protein on the blot (in the range of 10–70 μg of HeLa lysate protein loaded per lane; Fig. 2), we tested the correlation of lane density with actual protein load. HeLa cell lysate samples of known concentration with spiked-in ADH were separated using stain-free gels and then imaged to verify uniform loading and separation prior to blotting (Fig. 5a, inset). The proteins were then transferred to PVDF membranes and the blots were imaged using the stain-free imaging application on the ChemiDoc MP prior to antibody incubation to validate transfer efficiency, and to assure complete protein transfer to the membrane (Fig. 5a). We then compared the relative total lane density from the stain-free blot image with the relative μg quantity of HeLa lysate protein load (Fig. 5a), and found a positive Pearson Correlation (*p* value of 0.0398) supporting this method as a valid approach to normalize western blot data.

**Fig. 6:**
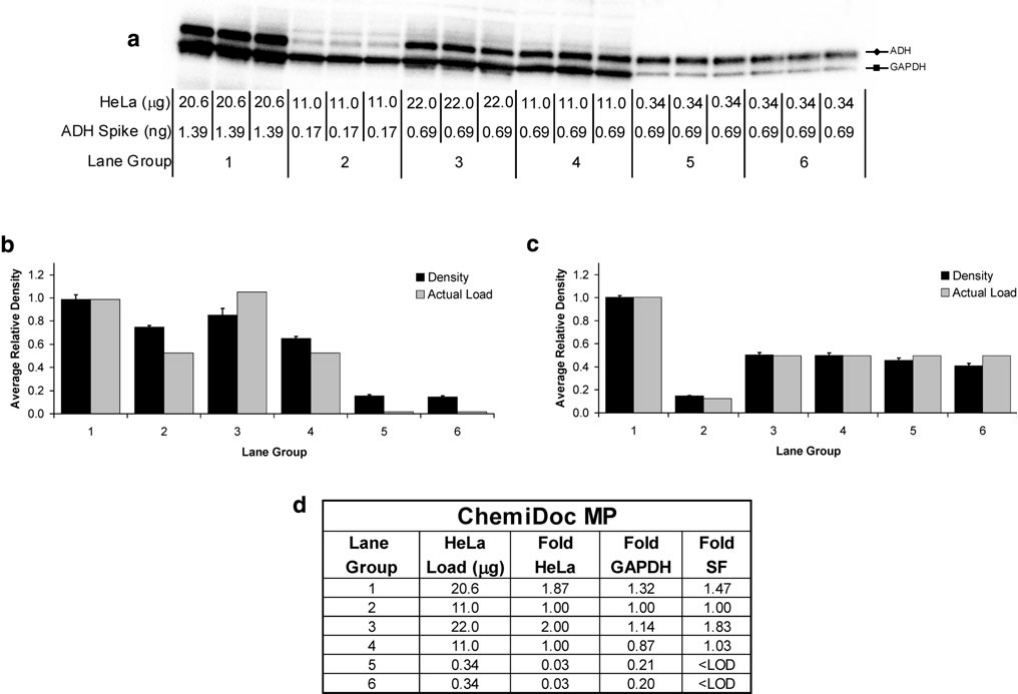
Densitometric analysis of protein bands imaged with the ChemiDoc MP. Quadruplicate chemiluminescent blots (**a**) were produced after stain-free image analysis (Fig. 5a). Blotting was performed using a mixture of rabbit- and mouse-derived primary antibodies to ADH and GAPDH, respectively, with the associated mixture of HRP-conjugated secondary antibodies. The average relative density of GAPDH (**b**) and ADH (**c**) was compared to the relative difference in μg quantity of protein load for the HeLa lysate (**b**) and the ng quantity of ADH-spike (**c**) between the triplicate replicates within each lane group over the four blots. The fold difference in stain-free (SF) detected lane density for total protein and GAPDH was compared to that of the μg quantity of actual loading of HeLa lysate (**d**). A positive Pearson Correlation was obtained for total protein (SF) but not for GAPDH (*p* values of 0.0398 and 0.155) **d**. <LOD—below limit of detection

**Fig. 7:**
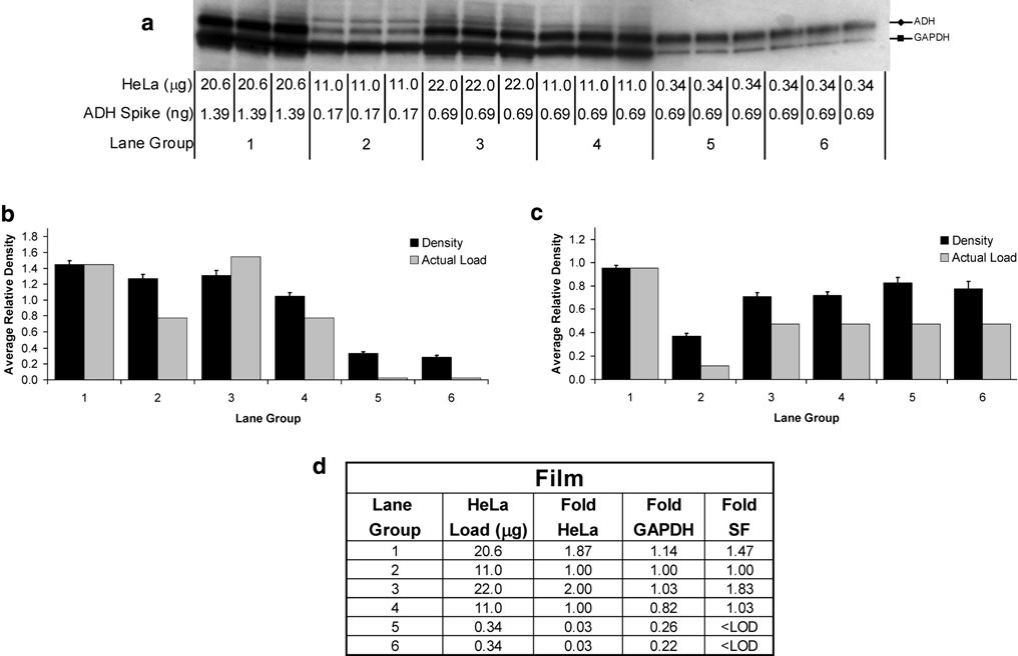
Densitometric analysis of blots imaged with film. The same four chemiluminescent blots from Fig. 6 were then imaged with film (**a**). Blotting was performed using a mixture of rabbit- and mouse-derived primary antibodies to ADH and GAPDH, respectively, with the associated mixture of HRP-conjugated secondary antibodies. The average relative density of GAPDH (**b**) and ADH (**c**) was compared to the relative difference in μg quantity of protein load for the HeLa lysate (**b**) and the ng quantity of ADH-spike (**c**) between the triplicate replicates within each lane group over four blots. The fold difference in stain-free (SF) detected lane density for total protein and GAPDH was compared to that of the μg quantity of actual loading of HeLa lysate (**d**). A positive Pearson Correlation was obtained for total protein (SF) but not for GAPDH (*p*-values of 0.0398 and 0.274 respectively). **d**. <LOD—below limit of detection

The relative intensities of the GAPDH bands were approximately equal among the lane groups 1–4 for both image-based (Fig. 6) and film-based (Fig. 7) detection. This data did not correlate well with the two-fold relative difference in μg quantity of HeLa lysate protein load yielding a negative Pearson Correlation (Figs. 6, 7, panels B and D). In contrast, the total lane density of transferred protein on the blots produced a better correlation with the fold change in protein load for the same lane groups (1–4), with a positive Pearson Correlation (*p* value of 0.0398) (Fig. 5b). This can be explained by contrasting the linear dynamic range for GAPDH and total protein, where at protein loads above about 0.5 μg, GAPDH is saturated (Fig. 3), and in the plateau whereas total protein lane density on the transferred blot is within the linear range of 10–70 μg (Fig. 2d). For lane groups 1–4, the protein loads between 11 and 22 μg, were well above the linear dynamic range of GAPDH but within that of the stain-free detection explaining much better quantification for the latter.

The HeLa lysate total protein load of 0.34 μg (lane groups 5 and 6) was well below the dynamic range and even below the detectable limit for stain-free imaging (Fig. 5a), but within the linear dynamic range for GAPDH (Fig. 3). This makes GAPDH an ideal loading control for highly abundant proteins which require much lower sample loading. It is worth pointing out that in these cases, stain-free imaging cannot be used for normalization. Care must be taken to ensure that the amount of lysate loaded in this case is within the linear dynamic range of both the loading control and the target protein to ensure accurate, quantifiable, and normalized densitometric data.

### Accurate Quantitation Using the Linear Dynamic Range

The 0.03-fold difference in HeLa lysate loading among lane groups 2, 5, and 6 (i.e., between 11 and 0.34 μg) was calculated to be only about 0.20–0.26-fold by relative band density of GAPDH (Figs. 6, 7, panel D contrast lane group 2 with 5 and 6 for “Fold GAPDH”). This can be explained by the fact that the total protein load of 0.34 μg is within the linear dynamic range of density for GAPDH but is in the plateau at 11 μg (Fig. 3). Thus, the densitometric fold difference is much smaller than expected because the two points are not within the linear range. This further underscores the importance of ensuring that the samples are diluted such that the loading control is well within the linear dynamic range of detection.

For ADH, the correlation between relative density and fold difference in ng quantity of spiked-in ADH protein load between all the lane groups was excellent when using the ChemiDoc MP imager (Fig. 6c), but poor with film (Fig. 7c). This is due to the different linear dynamic ranges obtained between film and the ChemiDoc MP for ADH (Fig. 4). All the loaded amounts of ADH (i.e., 0.17–1.39 ng) were within the linear dynamic range of detection for the ChemiDoc MP (Fig. 4b). However, this was not the case for the film, where the relative densities for ADH were measured from one value within the linear dynamic range (0.17 ng) and two values within the plateau (0.69 and 1.39 ng) (Figs. 3, 4).

## Conclusion

Accurate quantitation of relative protein expression is possible only by following appropriate experimental procedures to determine the linear and quantitative dynamic range for each target protein under a given set of experimental conditions. We show that only by producing a two-fold dilution series of the protein lysate can the linear range of quantitation be determined, and this is entirely dependent on the abundance of each target or loading control protein in the sample. Thus, each antibody in a given study should first be tested with a dilution series of a pooled protein lysate from the study samples. This will ensure that the appropriate dilutions of samples are used for accurate and normalized quantitation of the target proteins by means of densitometric analysis.

Film does not always offer the dynamic range to quantify the full range of protein expression among samples, and this can become further complicated by the need to objectively determine the saturation point of the target protein bands. The advent of cooled CCD camera technologies has permitted the automated and precise determination of the camera CCD saturation point of chemiluminescence, and permits a broader dynamic range than possible with film. Coupled with the appropriate use of sophisticated software analysis tools, accurate densitometric analysis of western blots is indeed possible.

Since many of the traditionally used loading controls are highly abundant and usually loaded in saturating quantities in the typical range of sample loading (10–70 μg per lane), they cannot be used for accurate normalization. Since many labs are publishing small changes (between two- and four-fold) among samples from western blots, accurate normalization becomes critical to ensure that the reported changes are real. The use of stain-free gel technology permits accurate normalization in the range of 10–70 μg of the total protein load, and therefore provides an excellent solution for normalization between the lanes of western blots by total protein transferred.

To conclude, we propose a rigorous methodology of validating sample loading, standardizing antibody dilutions, determining the dynamic range with a sensitive, camera-based imaging system, and use of a stain-free technology to get high-quality and reliable quantitative data from western blots.
